# *N*′-(2,4-Dichloro­benzyl­idene)-3-methoxy­benzohydrazide

**DOI:** 10.1107/S1600536810009645

**Published:** 2010-03-20

**Authors:** Chong-Gui Ren

**Affiliations:** a Department of Chemistry and Chemical Engineering, Zaozhuang University, Zaozhuang Shandong 277160, People’s Republic of China

## Abstract

There are two independent mol­ecules in the asymmetric unit of the title compound, C_15_H_12_Cl_2_N_2_O_2_. The dihedral angle between the two benzene rings is 27.6 (4)° in one mol­ecule and 16.4 (4)° in the other. Both mol­ecules adopt an *E* configuration about the C=N bonds. In the crystal structure, mol­ecules are linked through inter­molecular N—H⋯O hydrogen bonds, forming chains in the *a*-axis direction.

## Related literature

For the biological properties of Schiff base compounds, see: Jeewoth *et al.* (1999 ▶); Ren *et al.* (2002 ▶); Eltayeb *et al.* (2008 ▶); Sinha *et al.* (2008 ▶). For the structures of related Schiff bases previously reported by the author, see: Ren (2009*a* ▶,*b* ▶). For related structures, see: Cui *et al.* (2007 ▶); Jing *et al.* (2007 ▶); Ma *et al.* (2008 ▶); Salhin *et al.* (2007 ▶); Lin *et al.* (2007 ▶); Alhadi *et al.* (2008 ▶); Xue *et al.* (2008 ▶); Wang *et al.* (2008 ▶); Lu (2008 ▶); Diao *et al.* (2008 ▶); Qiu (2009 ▶); Mohd Lair *et al.* (2009*a* ▶,*b* ▶). For reference structural data, see: Allen *et al.* (1987 ▶).
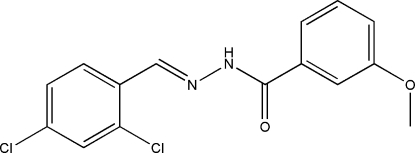


## Experimental

### 

#### Crystal data

C_15_H_12_Cl_2_N_2_O_2_
*M* *_r_* = 323.17
Triclinic, 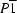

*a* = 8.298 (1) Å
*b* = 12.988 (2) Å
*c* = 14.138 (2) Å
α = 88.746 (3)°
β = 87.711 (2)°
γ = 84.020 (3)°
*V* = 1514.0 (4) Å^3^
*Z* = 4
Mo *K*α radiation
μ = 0.43 mm^−1^
*T* = 298 K
0.10 × 0.08 × 0.08 mm

#### Data collection

Bruker SMART CCD area-detector diffractometer
Absorption correction: multi-scan (*SADABS*; Sheldrick, 1996 ▶) *T*_min_ = 0.958, *T*_max_ = 0.966
6065 measured reflections
4120 independent reflections
2565 reflections with *I* > 2σ(*I*)
*R*_int_ = 0.024
θ_max_ = 22.9°

#### Refinement

*R*[*F* ^2^ > 2σ(*F* ^2^)] = 0.047
*wR*(*F* ^2^) = 0.121
*S* = 1.02
4120 reflections
381 parameters
H-atom parameters constrained
Δρ_max_ = 0.18 e Å^−3^
Δρ_min_ = −0.21 e Å^−3^

### 

Data collection: *SMART* (Bruker, 2002 ▶); cell refinement: *SAINT* (Bruker, 2002 ▶); data reduction: *SAINT*; program(s) used to solve structure: *SHELXS97* (Sheldrick, 2008 ▶); program(s) used to refine structure: *SHELXL97* (Sheldrick, 2008 ▶); molecular graphics: *SHELXTL* (Sheldrick, 2008 ▶); software used to prepare material for publication: *SHELXL97*.

## Supplementary Material

Crystal structure: contains datablocks global, I. DOI: 10.1107/S1600536810009645/sj2748sup1.cif

Structure factors: contains datablocks I. DOI: 10.1107/S1600536810009645/sj2748Isup2.hkl

Additional supplementary materials:  crystallographic information; 3D view; checkCIF report

## Figures and Tables

**Table 1:** Hydrogen-bond geometry (Å, °)

*D*—H⋯*A*	*D*—H	H⋯*A*	*D*⋯*A*	*D*—H⋯*A*
N4—H4*A*⋯O1^i^	0.86	2.02	2.844 (4)	161
N1—H1⋯O4^ii^	0.86	2.06	2.880 (3)	159

Symmetry codes: (i) 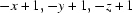; (ii) 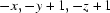.

**Fig. 1.**
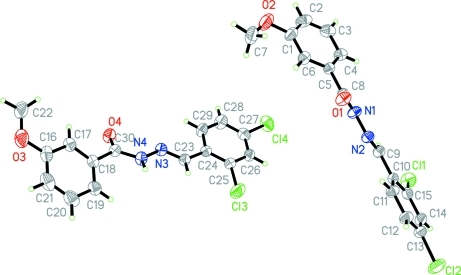
The molecular structure of the compound with 30% probability ellipsoids.

**Fig. 2.**
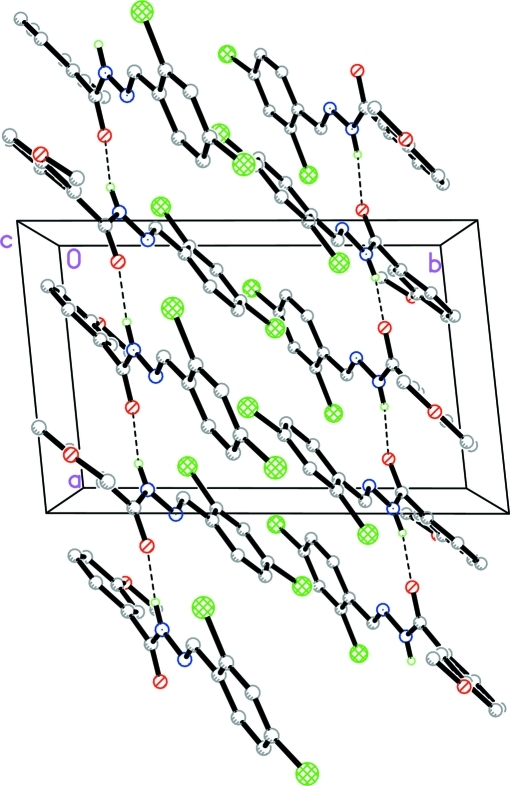
Molecular packing of the compound with hydrogen bonds drawn as dashed lines. Hydrogen atoms not involved in hydrogen bonding have been omitted.

## supplementary crystallographic information

### Comment

The Schiff base compounds show excellent biological properties (Jeewoth *et al.*, 1999; Ren *et al.*, 2002; Eltayeb *et al.*, 2008; Sinha *et al.*, 2008). Recently, the author has reported a few Schiff bases (Ren, 2009a,b). In this paper, the title new Schiff base compound is reported.

In the title compound, Fig. 1, the dihedral angle between the two benzene rings is 27.6 (4)° in one molecule and 16.4 (4)° in another molecule. All the bond lengths are within normal values (Allen *et al.*, 1987) and comparable to those in other similar compounds (Cui *et al.*, 2007; Jing *et al.*, 2007; Ma *et al.*, 2008; Salhin *et al.*, 2007; Lin *et al.*, 2007; Alhadi *et al.*, 2008; Xue *et al.*, 2008; Wang *et al.*, 2008; Lu, 2008; Diao *et al.*, 2008; Qiu, 2009; Mohd Lair *et al.*, 2009a,b).

In the crystal structure, molecules are linked through intermolecular N–H···O and O–H···O hydrogen bonds (Table 1), forming chains toward the *a* direction (Fig. 2).

### Experimental

All the starting materials were obtained with AR grade from Lancaster. 2,4-Dichlorobenzaldehyde (1.0 mmol, 174.0 mg) and 3-methoxybenzohydrazide (1.0 mmol, 166.2 mg) were refluxed in a 30 ml me thanol solution for 30 min to give a clear colorless solution. Colorless needle-shaped single crystals of the compound were obtained by slow evaporation of the solution for a week at room temperature.

### Refinement

H atoms were constrained to ideal geometries, with d(C–H) = 0.93 Å, d(N–H) = 0.86 Å, and with *U*_iso_(H) = 1.2*U*_eq_(C,*N*) and 1.5*U*_eq_(C_methyl_). The resolution is 22.92°, and the sin(theta)/Lambda < 0.6, which is caused by the weak diffraction of the crystal.

### Crystal data

**Table:**

C_15_H_12_Cl_2_N_2_O_2_	*Z* = 4
*M**_r_* = 323.17	*F*(000) = 664
Triclinic, *P*1	*D*_x_ = 1.418 Mg m^−^^3^
*a* = 8.298 (1) Å	Mo *K*α radiation, λ = 0.71073 Å
*b* = 12.988 (2) Å	Cell parameters from 1156 reflections
*c* = 14.138 (2) Å	θ = 2.8–23.5°
α = 88.746 (3)°	µ = 0.43 mm^−^^1^
β = 87.711 (2)°	*T* = 298 K
γ = 84.020 (3)°	Block, colorless
*V* = 1514.0 (4) Å^3^	0.10 × 0.08 × 0.08 mm

### Data collection

**Table:**

Bruker SMART CCD area-detector diffractometer	4120 independent reflections
Radiation source: fine-focus sealed tube	2565 reflections with *I* > 2σ(*I*)
graphite	*R*_int_ = 0.024
ω scans	θ_max_ = 22.9°, θ_min_ = 2.1°
Absorption correction: multi-scan (*SADABS*; Sheldrick, 1996)	*h* = −9→9
*T*_min_ = 0.958, *T*_max_ = 0.966	*k* = −10→14
6065 measured reflections	*l* = −15→15

### Refinement

**Table:**

Refinement on *F*^2^	Primary atom site location: structure-invariant direct methods
Least-squares matrix: full	Secondary atom site location: difference Fourier map
*R*[*F*^2^ > 2σ(*F*^2^)] = 0.047	Hydrogen site location: inferred from neighbouring sites
*wR*(*F*^2^) = 0.121	H-atom parameters constrained
*S* = 1.02	*w* = 1/[σ^2^(*F*_o_^2^) + (0.0469*P*)^2^ + 0.2383*P*] where *P* = (*F*_o_^2^ + 2*F*_c_^2^)/3
4120 reflections	(Δ/σ)_max_ = 0.001
381 parameters	Δρ_max_ = 0.18 e Å^−^^3^
0 restraints	Δρ_min_ = −0.21 e Å^−^^3^

### Special details

**Table:**

Geometry. All esds (except the esd in the dihedral angle between two l.s. planes) are estimated using the full covariance matrix. The cell esds are taken into account individually in the estimation of esds in distances, angles and torsion angles; correlations between esds in cell parameters are only used when they are defined by crystal symmetry. An approximate (isotropic) treatment of cell esds is used for estimating esds involving l.s. planes.
Refinement. Refinement of *F*^2^ against ALL reflections. The weighted *R*-factor wR and goodness of fit *S* are based on *F*^2^, conventional *R*-factors *R* are based on *F*, with *F* set to zero for negative *F*^2^. The threshold expression of *F*^2^ > σ(*F*^2^) is used only for calculating *R*-factors(gt) etc. and is not relevant to the choice of reflections for refinement. *R*-factors based on *F*^2^ are statistically about twice as large as those based on *F*, and *R*- factors based on ALL data will be even larger.

### Fractional atomic coordinates and isotropic or equivalent isotropic displacement parameters (Å^2^)

**Table:**

	*x*	*y*	*z*	*U*_iso_*/*U*_eq_	
Cl1	0.30439 (13)	0.32267 (9)	1.09237 (7)	0.0867 (4)	
Cl2	0.83230 (16)	0.50607 (10)	1.16976 (8)	0.1062 (5)	
Cl3	0.1167 (2)	0.70669 (11)	0.60769 (9)	0.1330 (6)	
Cl4	−0.35105 (15)	0.45751 (9)	0.67302 (8)	0.1007 (4)	
N1	0.4406 (3)	0.2114 (2)	0.75587 (19)	0.0555 (8)	
H1	0.3412	0.2033	0.7709	0.067*	
N2	0.5340 (3)	0.2588 (2)	0.8166 (2)	0.0531 (7)	
N3	−0.0370 (3)	0.7340 (2)	0.3205 (2)	0.0556 (8)	
N4	0.0573 (3)	0.7900 (2)	0.2608 (2)	0.0574 (8)	
H4A	0.1500	0.8056	0.2777	0.069*	
O1	0.6495 (3)	0.18861 (18)	0.64745 (16)	0.0631 (7)	
O2	0.3350 (4)	0.1097 (3)	0.3553 (2)	0.1031 (10)	
O3	0.2072 (4)	0.9215 (3)	−0.1371 (2)	0.1031 (10)	
O4	−0.1296 (3)	0.79782 (19)	0.14806 (16)	0.0669 (7)	
C1	0.3271 (5)	0.0976 (3)	0.4518 (3)	0.0720 (12)	
C2	0.2182 (5)	0.0314 (3)	0.4875 (4)	0.0827 (14)	
H2	0.1565	−0.0014	0.4462	0.099*	
C3	0.2003 (5)	0.0137 (3)	0.5828 (3)	0.0762 (12)	
H3	0.1272	−0.0313	0.6057	0.091*	
C4	0.2901 (4)	0.0622 (3)	0.6454 (3)	0.0595 (10)	
H4	0.2764	0.0513	0.7103	0.071*	
C5	0.4007 (4)	0.1271 (2)	0.6100 (2)	0.0472 (8)	
C6	0.4199 (4)	0.1441 (3)	0.5135 (2)	0.0593 (10)	
H6	0.4959	0.1870	0.4903	0.071*	
C7	0.4439 (7)	0.1770 (4)	0.3170 (3)	0.121 (2)	
H7A	0.4126	0.2458	0.3391	0.181*	
H7B	0.4420	0.1769	0.2492	0.181*	
H7C	0.5514	0.1541	0.3366	0.181*	
C8	0.5085 (4)	0.1778 (3)	0.6720 (2)	0.0502 (9)	
C9	0.4671 (4)	0.2843 (3)	0.8961 (2)	0.0542 (9)	
H9	0.3617	0.2695	0.9109	0.065*	
C10	0.5559 (4)	0.3368 (2)	0.9639 (2)	0.0496 (9)	
C11	0.7065 (4)	0.3690 (3)	0.9398 (2)	0.0573 (10)	
H11	0.7503	0.3566	0.8790	0.069*	
C12	0.7937 (4)	0.4184 (3)	1.0020 (3)	0.0647 (10)	
H12	0.8955	0.4376	0.9840	0.078*	
C13	0.7290 (5)	0.4392 (3)	1.0910 (3)	0.0660 (11)	
C14	0.5802 (5)	0.4090 (3)	1.1195 (3)	0.0677 (11)	
H14	0.5376	0.4222	1.1803	0.081*	
C15	0.4949 (4)	0.3583 (3)	1.0553 (3)	0.0568 (9)	
C16	0.1987 (5)	0.9281 (3)	−0.0409 (3)	0.0718 (11)	
C17	0.1040 (4)	0.8705 (3)	0.0187 (3)	0.0618 (10)	
H17	0.0383	0.8248	−0.0062	0.074*	
C18	0.1085 (4)	0.8818 (3)	0.1148 (3)	0.0525 (9)	
C19	0.2046 (4)	0.9498 (3)	0.1527 (3)	0.0704 (11)	
H19	0.2085	0.9561	0.2179	0.084*	
C20	0.2954 (5)	1.0084 (3)	0.0920 (4)	0.0891 (14)	
H20	0.3589	1.0556	0.1165	0.107*	
C21	0.2917 (5)	0.9971 (4)	−0.0028 (4)	0.0869 (14)	
H21	0.3532	1.0368	−0.0428	0.104*	
C22	0.1221 (7)	0.8470 (4)	−0.1775 (3)	0.1154 (19)	
H22A	0.1567	0.7799	−0.1511	0.173*	
H22B	0.1432	0.8467	−0.2447	0.173*	
H22C	0.0080	0.8632	−0.1642	0.173*	
C23	0.0139 (4)	0.7167 (3)	0.4036 (3)	0.0564 (9)	
H23	0.1082	0.7429	0.4212	0.068*	
C24	−0.0752 (4)	0.6559 (3)	0.4711 (2)	0.0527 (9)	
C25	−0.0405 (5)	0.6459 (3)	0.5646 (3)	0.0659 (10)	
C26	−0.1259 (5)	0.5868 (3)	0.6285 (3)	0.0726 (12)	
H26	−0.1005	0.5820	0.6920	0.087*	
C27	−0.2481 (5)	0.5360 (3)	0.5956 (3)	0.0683 (11)	
C28	−0.2864 (5)	0.5438 (3)	0.5027 (3)	0.0778 (12)	
H28	−0.3698	0.5090	0.4810	0.093*	
C29	−0.2012 (4)	0.6033 (3)	0.4413 (3)	0.0674 (11)	
H29	−0.2285	0.6087	0.3781	0.081*	
C30	0.0012 (4)	0.8200 (3)	0.1750 (3)	0.0531 (9)	

### Atomic displacement parameters (Å^2^)

**Table:**

	*U*^11^	*U*^22^	*U*^33^	*U*^12^	*U*^13^	*U*^23^
Cl1	0.0859 (8)	0.1052 (9)	0.0706 (7)	−0.0250 (7)	0.0176 (6)	−0.0033 (6)
Cl2	0.1145 (10)	0.1176 (10)	0.0918 (9)	−0.0174 (8)	−0.0375 (7)	−0.0345 (7)
Cl3	0.1856 (15)	0.1449 (12)	0.0878 (9)	−0.0930 (11)	−0.0557 (9)	0.0140 (8)
Cl4	0.1080 (10)	0.1002 (9)	0.0905 (9)	−0.0132 (7)	0.0349 (7)	0.0183 (7)
N1	0.0392 (17)	0.075 (2)	0.0551 (19)	−0.0155 (16)	−0.0011 (15)	−0.0138 (16)
N2	0.0478 (18)	0.0611 (19)	0.0521 (19)	−0.0122 (15)	−0.0036 (15)	−0.0047 (15)
N3	0.0415 (18)	0.067 (2)	0.058 (2)	−0.0088 (15)	0.0041 (16)	0.0035 (16)
N4	0.0361 (17)	0.077 (2)	0.061 (2)	−0.0155 (16)	−0.0023 (15)	0.0086 (16)
O1	0.0404 (15)	0.0914 (19)	0.0610 (16)	−0.0221 (14)	0.0026 (12)	−0.0127 (13)
O2	0.113 (3)	0.130 (3)	0.065 (2)	0.007 (2)	−0.0319 (18)	−0.015 (2)
O3	0.123 (3)	0.104 (3)	0.075 (2)	0.006 (2)	0.031 (2)	0.0132 (19)
O4	0.0465 (16)	0.095 (2)	0.0618 (16)	−0.0221 (14)	−0.0070 (13)	0.0059 (14)
C1	0.069 (3)	0.088 (3)	0.055 (3)	0.018 (3)	−0.019 (2)	−0.019 (2)
C2	0.053 (3)	0.095 (3)	0.102 (4)	−0.002 (3)	−0.029 (3)	−0.035 (3)
C3	0.053 (3)	0.076 (3)	0.102 (4)	−0.014 (2)	−0.005 (2)	−0.017 (3)
C4	0.042 (2)	0.068 (2)	0.069 (3)	−0.007 (2)	−0.0035 (19)	−0.011 (2)
C5	0.038 (2)	0.054 (2)	0.049 (2)	−0.0020 (18)	−0.0058 (16)	−0.0042 (17)
C6	0.052 (2)	0.068 (3)	0.057 (2)	−0.001 (2)	−0.0038 (19)	−0.005 (2)
C7	0.180 (6)	0.118 (4)	0.058 (3)	0.020 (4)	−0.016 (3)	0.007 (3)
C8	0.045 (2)	0.056 (2)	0.050 (2)	−0.0073 (19)	−0.0034 (18)	−0.0003 (18)
C9	0.045 (2)	0.062 (2)	0.056 (2)	−0.0084 (19)	−0.0048 (18)	−0.0019 (19)
C10	0.051 (2)	0.049 (2)	0.048 (2)	−0.0026 (18)	−0.0083 (18)	−0.0001 (17)
C11	0.048 (2)	0.065 (2)	0.059 (2)	−0.004 (2)	−0.0059 (19)	−0.0113 (19)
C12	0.057 (2)	0.070 (3)	0.069 (3)	−0.011 (2)	−0.009 (2)	−0.006 (2)
C13	0.071 (3)	0.058 (2)	0.070 (3)	−0.001 (2)	−0.023 (2)	−0.010 (2)
C14	0.086 (3)	0.066 (3)	0.050 (2)	0.004 (2)	−0.011 (2)	−0.006 (2)
C15	0.060 (2)	0.053 (2)	0.056 (2)	−0.0006 (19)	−0.0043 (19)	0.0035 (19)
C16	0.065 (3)	0.079 (3)	0.066 (3)	0.012 (2)	0.011 (2)	0.012 (2)
C17	0.052 (2)	0.066 (3)	0.066 (3)	−0.003 (2)	0.008 (2)	0.004 (2)
C18	0.038 (2)	0.055 (2)	0.062 (3)	0.0000 (18)	0.0044 (18)	0.0057 (19)
C19	0.057 (3)	0.075 (3)	0.082 (3)	−0.018 (2)	−0.010 (2)	0.012 (2)
C20	0.069 (3)	0.085 (3)	0.117 (4)	−0.027 (3)	−0.017 (3)	0.019 (3)
C21	0.052 (3)	0.094 (4)	0.113 (4)	−0.014 (3)	0.002 (3)	0.031 (3)
C22	0.180 (6)	0.099 (4)	0.059 (3)	0.010 (4)	0.023 (3)	−0.003 (3)
C23	0.050 (2)	0.063 (2)	0.058 (2)	−0.0098 (19)	−0.004 (2)	0.0012 (19)
C24	0.049 (2)	0.054 (2)	0.055 (2)	−0.0016 (19)	0.0003 (18)	−0.0036 (18)
C25	0.082 (3)	0.060 (2)	0.057 (3)	−0.012 (2)	−0.010 (2)	−0.002 (2)
C26	0.103 (4)	0.066 (3)	0.047 (2)	−0.002 (3)	−0.001 (2)	0.001 (2)
C27	0.067 (3)	0.063 (3)	0.072 (3)	−0.002 (2)	0.024 (2)	−0.001 (2)
C28	0.055 (3)	0.108 (3)	0.071 (3)	−0.020 (2)	0.007 (2)	0.004 (3)
C29	0.054 (2)	0.098 (3)	0.051 (2)	−0.016 (2)	0.002 (2)	0.003 (2)
C30	0.040 (2)	0.061 (2)	0.057 (2)	−0.0032 (19)	0.0033 (19)	−0.0022 (19)

### Geometric parameters (Å, °)

**Table:**

Cl1—C15	1.750 (4)	C10—C11	1.387 (4)
Cl2—C13	1.733 (4)	C10—C15	1.392 (4)
Cl3—C25	1.730 (4)	C11—C12	1.371 (4)
Cl4—C27	1.739 (4)	C11—H11	0.9300
N1—C8	1.353 (4)	C12—C13	1.369 (5)
N1—N2	1.376 (3)	C12—H12	0.9300
N1—H1	0.8600	C13—C14	1.377 (5)
N2—C9	1.268 (4)	C14—C15	1.390 (5)
N3—C23	1.272 (4)	C14—H14	0.9300
N3—N4	1.376 (3)	C16—C21	1.374 (5)
N4—C30	1.351 (4)	C16—C17	1.389 (5)
N4—H4A	0.8600	C17—C18	1.372 (5)
O1—C8	1.229 (3)	C17—H17	0.9300
O2—C1	1.369 (4)	C18—C19	1.380 (5)
O2—C7	1.406 (5)	C18—C30	1.490 (4)
O3—C16	1.363 (4)	C19—C20	1.387 (5)
O3—C22	1.400 (5)	C19—H19	0.9300
O4—C30	1.229 (4)	C20—C21	1.354 (6)
C1—C6	1.376 (5)	C20—H20	0.9300
C1—C2	1.385 (6)	C21—H21	0.9300
C2—C3	1.366 (5)	C22—H22A	0.9600
C2—H2	0.9300	C22—H22B	0.9600
C3—C4	1.382 (5)	C22—H22C	0.9600
C3—H3	0.9300	C23—C24	1.454 (4)
C4—C5	1.382 (4)	C23—H23	0.9300
C4—H4	0.9300	C24—C25	1.366 (5)
C5—C6	1.382 (4)	C24—C29	1.390 (5)
C5—C8	1.488 (4)	C25—C26	1.392 (5)
C6—H6	0.9300	C26—C27	1.367 (5)
C7—H7A	0.9600	C26—H26	0.9300
C7—H7B	0.9600	C27—C28	1.363 (5)
C7—H7C	0.9600	C28—C29	1.374 (5)
C9—C10	1.455 (4)	C28—H28	0.9300
C9—H9	0.9300	C29—H29	0.9300
			
C8—N1—N2	118.6 (3)	C13—C14—H14	120.7
C8—N1—H1	120.7	C15—C14—H14	120.7
N2—N1—H1	120.7	C14—C15—C10	122.0 (3)
C9—N2—N1	116.1 (3)	C14—C15—Cl1	117.8 (3)
C23—N3—N4	116.0 (3)	C10—C15—Cl1	120.2 (3)
C30—N4—N3	118.6 (3)	O3—C16—C21	116.1 (4)
C30—N4—H4A	120.7	O3—C16—C17	124.4 (4)
N3—N4—H4A	120.7	C21—C16—C17	119.4 (4)
C1—O2—C7	117.1 (4)	C18—C17—C16	119.3 (4)
C16—O3—C22	117.3 (4)	C18—C17—H17	120.4
O2—C1—C6	125.1 (4)	C16—C17—H17	120.4
O2—C1—C2	115.8 (4)	C17—C18—C19	121.0 (3)
C6—C1—C2	119.1 (4)	C17—C18—C30	116.8 (3)
C3—C2—C1	120.7 (4)	C19—C18—C30	122.2 (3)
C3—C2—H2	119.6	C18—C19—C20	119.0 (4)
C1—C2—H2	119.6	C18—C19—H19	120.5
C2—C3—C4	120.5 (4)	C20—C19—H19	120.5
C2—C3—H3	119.7	C21—C20—C19	120.1 (4)
C4—C3—H3	119.7	C21—C20—H20	119.9
C3—C4—C5	118.9 (4)	C19—C20—H20	119.9
C3—C4—H4	120.5	C20—C21—C16	121.2 (4)
C5—C4—H4	120.5	C20—C21—H21	119.4
C6—C5—C4	120.5 (3)	C16—C21—H21	119.4
C6—C5—C8	117.1 (3)	O3—C22—H22A	109.5
C4—C5—C8	122.4 (3)	O3—C22—H22B	109.5
C1—C6—C5	120.2 (4)	H22A—C22—H22B	109.5
C1—C6—H6	119.9	O3—C22—H22C	109.5
C5—C6—H6	119.9	H22A—C22—H22C	109.5
O2—C7—H7A	109.5	H22B—C22—H22C	109.5
O2—C7—H7B	109.5	N3—C23—C24	120.0 (3)
H7A—C7—H7B	109.5	N3—C23—H23	120.0
O2—C7—H7C	109.5	C24—C23—H23	120.0
H7A—C7—H7C	109.5	C25—C24—C29	116.8 (3)
H7B—C7—H7C	109.5	C25—C24—C23	123.1 (3)
O1—C8—N1	122.8 (3)	C29—C24—C23	120.1 (3)
O1—C8—C5	121.5 (3)	C24—C25—C26	122.5 (4)
N1—C8—C5	115.7 (3)	C24—C25—Cl3	120.0 (3)
N2—C9—C10	119.8 (3)	C26—C25—Cl3	117.4 (3)
N2—C9—H9	120.1	C27—C26—C25	118.5 (4)
C10—C9—H9	120.1	C27—C26—H26	120.8
C11—C10—C15	116.5 (3)	C25—C26—H26	120.8
C11—C10—C9	121.1 (3)	C28—C27—C26	120.8 (4)
C15—C10—C9	122.4 (3)	C28—C27—Cl4	119.9 (4)
C12—C11—C10	122.6 (3)	C26—C27—Cl4	119.3 (3)
C12—C11—H11	118.7	C27—C28—C29	119.6 (4)
C10—C11—H11	118.7	C27—C28—H28	120.2
C13—C12—C11	119.2 (4)	C29—C28—H28	120.2
C13—C12—H12	120.4	C28—C29—C24	121.8 (4)
C11—C12—H12	120.4	C28—C29—H29	119.1
C12—C13—C14	121.0 (3)	C24—C29—H29	119.1
C12—C13—Cl2	120.4 (3)	O4—C30—N4	122.4 (3)
C14—C13—Cl2	118.6 (3)	O4—C30—C18	122.0 (3)
C13—C14—C15	118.6 (3)	N4—C30—C18	115.7 (3)

### Hydrogen-bond geometry (Å, °)

**Table:**

*D*—H···*A*	*D*—H	H···*A*	*D*···*A*	*D*—H···*A*
N4—H4A···O1^i^	0.86	2.02	2.844 (4)	161
N1—H1···O4^ii^	0.86	2.06	2.880 (3)	159

Symmetry codes: (i) −*x*+1, −*y*+1, −*z*+1; (ii) −*x*, −*y*+1, −*z*+1.
